# A case report about a child with drug-resistant tuberculous meningitis

**DOI:** 10.1186/s12879-023-07990-x

**Published:** 2023-02-07

**Authors:** Jing Tong, Mengqiu Gao, Yu Chen, Jie Wang

**Affiliations:** 1 Department of Tuberculosis, Beijing Chest Hospital, Capital Medical University, Beijing Tuberculosis and Thoracic Tumor Institute, Area 2, Yard 9, Beiguan Street, Yongzhun Town, Tongzhou District, Beijing, 101100 China; 2 Department of Tuberculosis, The Sixth People’s Hospital of Zhengzhou, Zhengzhou, China

**Keywords:** Tuberculosis, Tuberculous meningitis, Children, Drug resistance, Diagnosis, Treatment

## Abstract

**Background:**

Hematogenous disseminated tuberculosis predisposes to concurrent tuberculous meningitis (TBM), the most devastating and disabling form of tuberculosis. However, children often have atypical clinical symptoms, difficulty in specimen collection, low specimen content, and an increasing incidence of drug-resistant tuberculosis. Thus, the accurate diagnosis and timely treatment of childhood tuberculosis face monumental challenges.

**Case presentation:**

The 14-year-old female presented to the hospital with intermittent fever, headache, and blurred vision. Her cerebrospinal fluid (CSF) showed a lymphocytic pleocytosis, an elevated protein level, and a decreased chloride level. And her CSF tested positive for TB-RNA. Xpert MTB/RIF detected Mycobacterium tuberculosis in her CSF, but the rifampin resistance test was unknown. Subsequently, her CSF culture was positive for Mycobacterium tuberculosis. The drug sensitivity test (DST) revealed resistance to isoniazid, rifampin, and fluoroquinolones. A computed tomography (CT) of the chest showed diffuse miliary nodules in both lungs. Intracranial enhanced magnetic resonance imaging (MRI) showed “multiple intensified images of the brain parenchyma, cisterns, and part of the meninges.” The final diagnosis is miliary pulmonary tuberculosis and pre-extensive drug-resistant TBM. After 19 months of an oral, individualized antituberculosis treatment, she recovered with no significant neurological sequelae.

**Conclusion:**

For patients with miliary pulmonary tuberculosis, especially children, even if there are no typical clinical symptoms, it is necessary to know whether there is TBM and other conditions. Always look for the relevant aetiological basis to clarify whether it is drug-resistant tuberculosis. Only a rapid and accurate diagnosis and timely and effective treatment can improve the prognosis and reduce mortality and disability rates.

## Background

Tuberculosis (TB) is one of the world’s most serious diseases that endanger human health, especially among children. According to what the World Health Organization (WHO) reported in 2022, there were about 10.6 million TB patients worldwide in 2021, of whom 1.166 million were children. Globally, the estimated number of deaths from TB was 1.6 million, up from both 2019 and 2020, with about 217,000 children dead [1]. When Mycobacterium tuberculosis enters the bloodstream, it spreads widely to the lungs and causes lesions that become miliary pulmonary tuberculosis. And in severe cases, it can spread to multiple organs throughout the body. Tuberculous meningitis (TBM) is the most destructive and disabling form of tuberculosis in children and adolescents. However, as a special group, children often have atypical clinical symptoms, difficulty with specimen collection, low specimen content, limited testing conditions, etc. There is an increasing incidence of drug-resistant tuberculosis. According to WHO estimates, the number of drug-resistant tuberculosis patients in 2021 was 450,000, an increase of 3.1% over the 437,000 cases in 2020. The global burden of tuberculosis has further increased, making this population face many difficulties and challenges in diagnosis and treatment [2, 3]. In recent years, with the emergence of new technologies for tuberculosis detection and new treatment protocols, more and more patients, especially drug-resistant tuberculosis patients, have been diagnosed and treated promptly and have continuously achieved remarkable results. However, the reported data in the literature on drug-resistant tuberculous meningitis in children is limited. Here, we reported a case of the diagnosis and treatment of a child with miliary pulmonary tuberculosis and drug-resistant TBM.

## Case presentation

A 14-year-old girl, presented to the local hospital on July 6, 2019, with 5 days of intermittent fever and a maximum temperature of 38.5℃. She had intermittent right chest pain, without coughing, sputum production, or chest tightness. The local doctor gave her an anti-infective treatment for “pneumonia” for 7 days because of the patchy high-density lung shadow on her chest CT scan, but it did not help her condition. Then she presented to the local TB hospital on July 15, 2019. Here she got a diagnosis of “Mycobacterium tuberculosis-negative pulmonary tuberculosis” based on the chest CT findings, positive interferon-gamma release assay (IGRA) results, and positive tuberculin skin test (TST). The sputum acid-fast bacilli smear was negative. She started anti-tuberculosis medication at a dose of “0.3 g/day of isoniazid, 0.45 g/day of rifampin, 1.0 g/day of pyrazinamide, and 0.75 g/day of ethambutol.” After 2 months of treatment, her fever broke and her chest pain lessened. On October 16, 2019, she went to the hospital, and a chest CT revealed diffuse miliary nodules in both lungs. Her sputum acid-fast bacilli smear was still negative. She was currently receiving high-dose isoniazid (0.6 g/day) and prednisone acetate (30 mg/day) for miliary pulmonary tuberculosis. Prednisone acetate was subsequently tapered and discontinued. However, the youngster experienced a fever again on December 16, 2019, reaching a high of 38.8 °C without chills, a cough, or sputum. She also experienced a paroxysmal headache and blurred vision. Because of the worsening of her headache, she visited the hospital once more on December 30, 2019, and the cranial brain magnetic resonance imaging (MRI) revealed atypical intracranial lesions that were deemed to be TBM. In an emergency, she came to our hospital for further treatment. Prior medical history: no history of hepatitis, tuberculosis, malaria, hypertension, diabetes, cardiovascular disease, psychiatric illness, surgery, trauma, blood transfusion, or allergies. Denial of a history of close contact with active tuberculosis.

**Fig. 1:**
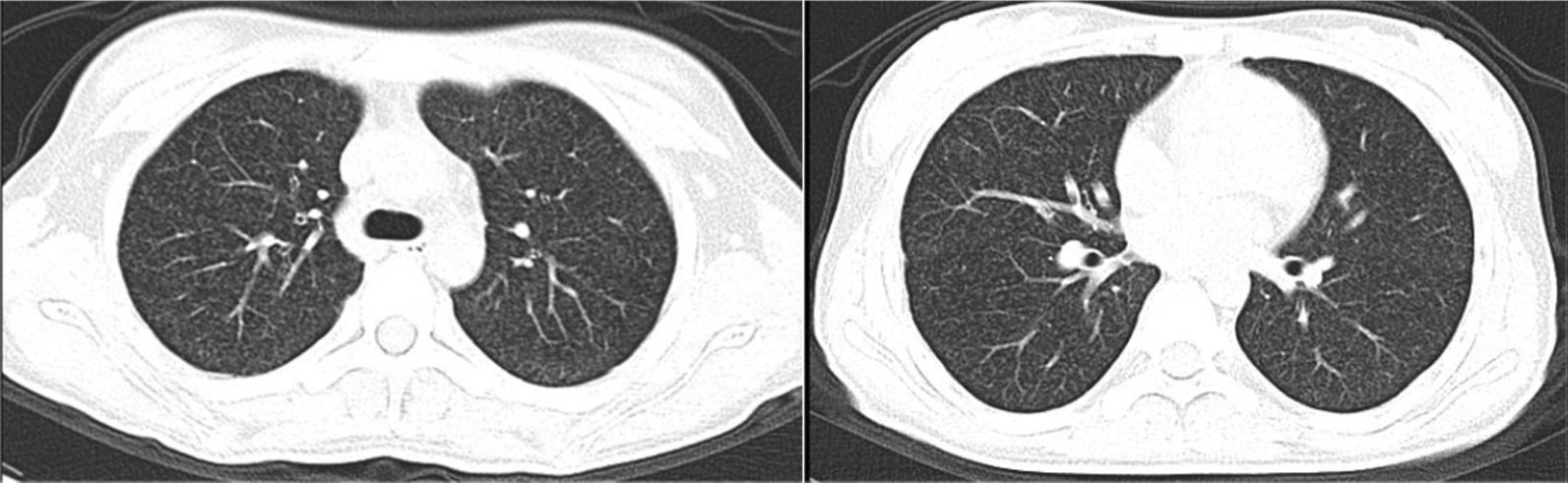
Representative slices of chest CT images showed diffuse miliary nodules in both lungs

**Fig. 2:**
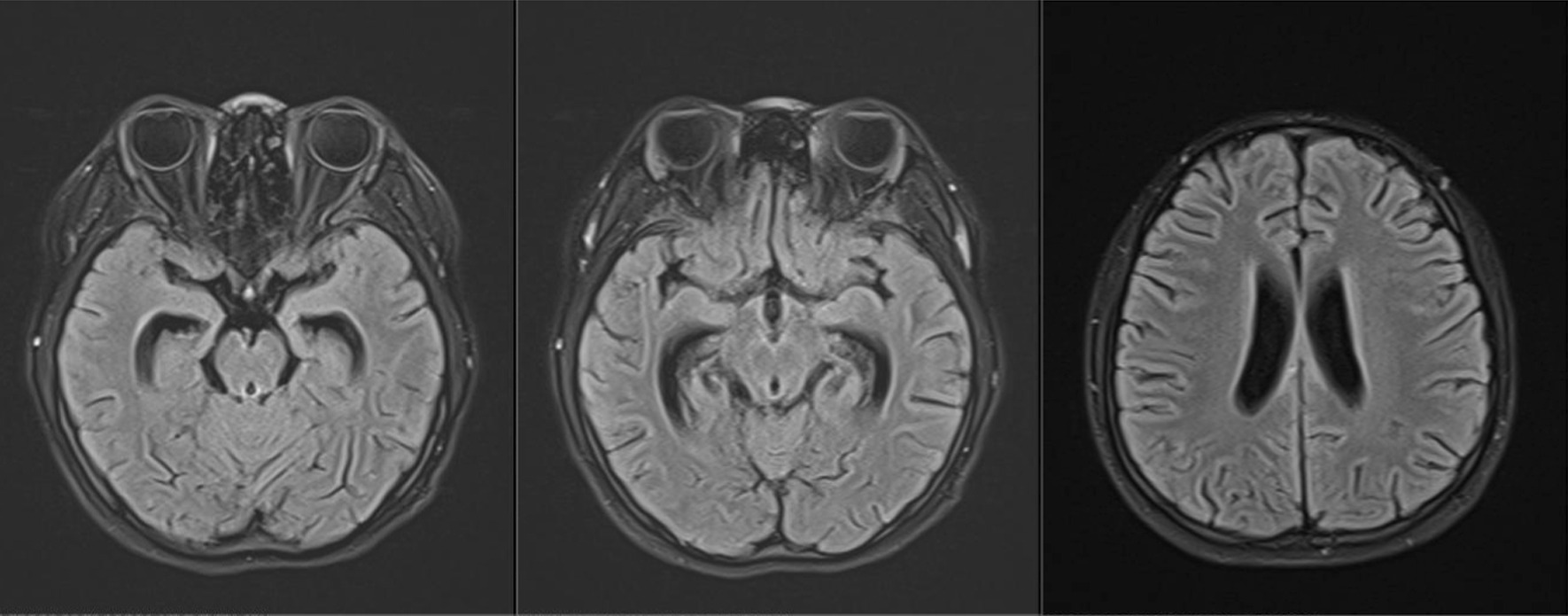
The cranial enhancement MRI showed punctiform enhancement in the pontine brain, right cerebellar hemisphere, bilateral frontal, temporal and parietal lobes, nodular enhancement in the local meninges, and linear enhancement in the brain pool

When arriving at our hospital, she was febrile (38.6℃). Her vital signs were a heart rate of 112 per minute, a respiratory rate of 24 per minute, blood pressure 109/68 mmHg, and oxygen saturation in room air of 98%. Her physical examination showed slight neck rigidity, positive Kerning's sign, and positive Brudzinski's sign. Her chest CT showed diffuse miliary nodules in both lungs (Fig. 1). The cranial enhancement MRI showed punctiform enhancement in the pontine brain, right cerebellar hemisphere, bilateral frontal, temporal, parietal lobes, nodular enhancement in the local meninges, and linear enhancement in the brain pool (Fig. 2). Considering the possibility of tuberculous meningitis, we immediately obtained a specimen of her CSF. On January 02, 2020, she had her first lumbar puncture. And a culture of Mycobacterium tuberculosis (liquid culture, medium MGIT 960) in the CSF was taken. Her other CSF results showed a lymphocytic pleocytosis, elevated protein level (1.33 g/L, normal value 0.08–0.43 g/L), decreased chloride level (116 mmol/L, normal value 118–132 mmol/L), normal glucose level (2.56 mmol/L, normal value 2.2–3.9 mmol/L), smear-negative for acid-fast bacilli, positive for TB-RNA, Xert MTB/RIF detected Mycobacterium tuberculosis, but rifampin resistance test was unknown. Her sputum acid-fast bacilli smear, TB-RNA, and Xpert were all negative. TBM was confirmed, but the rifampicin resistance was indeterminate. Her first rapid culture of CSF showed positive result on January 28, 2020. We then undertook the traditional-culture based phenotypic testing DST and continued treatment as sensitive TB while waiting for the DST result. The basic treatment regimen is "isoniazid 0.6 g/day, rifampin 0.45 g/day, pyrazinamide 1.0 g/day, and ethambutol 0.75 g/day". Also added "linezolid 0.6 g/day, prothioconazole 0.6 g/day, and prednisone acetate 30 mg/day", in order to enhance the efficacy and ease clinical symptoms. The child’s headache subsided, her body temperature gradually returned to normal, and her vision cleared. However, CSF protein was still higher than 1 g/L. Her chest CT scan revealed a substantial decrease in bilateral lung lesions on March 14, 2020. While her brain enhancement MRI showed punctiform enhancement in the left temporal lobe and right parietal lobe, and nodular-like significant enhancement in the left pontocerebellar horn region, with a slightly larger lesion than before. On March 20, 2020, the DST result showed resistance to “isoniazid, rifampin, streptomycin, levofloxacin, and moxifloxacin”. Finally, we diagnosed her with pre-extensive drug-resistant TBM (pre-XDR TBM).

After considering WHO guidelines for the diagnosis and treatment of drug-resistant TB, drug sensitivity results, the patient’s medication history, and drug penetration in the CSF, we developed an individualized treatment regimen for her. The regimen included linezolid (0.6 g/day), cycloserine (0.5 g/day), clofazimine (0.1 g/day), pyrazinamide (1.0 g/day), ethambutol (0.75 g/day), and prothionamide (0.6 g/day), with vitamin B6 (100 mg/day) and symptomatic supportive therapy. Three months after the therapy regimen changed, her brain MRI showed enlarged upper ocular chiasm, suprasellar cistern, and interpeduncular cistern lesions. Since there were no obvious signs of symptoms, she continued her treatment. In the sixth month of treatment in 2020.09, she developed numbness in both lower legs and feet, which could be tolerated. We confirmed it was “mild peripheral neuropathy” caused by “linezolid”, and advised her to continue to take nutritional neurological drugs such as B vitamins. After 7 months of treatment, the child’s CSF parameters returned to normal. However, she developed joint pain in the lower limbs and a uric acid test of 719 umol/L. We thought it was an adverse reaction to pyrazinamide, excluding other factors. The patient completed the intensive phase of treatment and was recovering well, so we discontinued both ethambutol and pyrazinamide. And the child’s symptoms were significantly relieved after discontinuation of the drug. The child experienced a decrease in visual acuity after 9 months of treatment. After excluding the loss of vision caused by tuberculosis and other factors, we considered it a side effect of linezolid, we discontinued it. After discontinuing linezolid for 1 week, her vision gradually returned to normal. She continued the treatment with the remaining three drugs, with no other significant adverse reactions throughout the treatment period. Throughout the treatment period, all sputum cultures from the patient were negative. Her CSF pressure, protein level, and cell counts continued to be normal after 7 months of treatment. At the completion of 19 months of treatment, the patient’s pulmonary and brain TB lesions had all been absorbed, so we discontinued her treatment. There were no neurological sequelae other than mild peripheral neuropathy.

## Discussion and conclusions

Tuberculosis remains one of the infectious diseases that threaten children’s health. Children are often different from adults in terms of onset, clinical manifestations, diagnosis, and treatment. Kids infected with tuberculosis bacteria are prone to involve multiple organs throughout the body, and hematogenous disseminated tuberculosis occurs, of which TBM is a severe and devastating type of tuberculosis that seriously threatens children’s lives [4, 5]. The youngster has atypical clinical symptoms, a low etiological positivity rate, difficulty with early diagnosis, and a top case fatality rate. More than half of the TBM survivors have neurological sequelae [6, 7]. Drug-resistant tuberculosis is becoming more and more common, making the diagnosis and treatment of drug-resistant TBM in children more torturous.

The WHO suggests the staff can use imaging as an evaluation for the diagnosis of tuberculosis [8]. When the medical staff has insufficient experience in tuberculosis or encounter diseases that are easily confused with others. It can delay the diagnosis and treatment of tuberculosis, just like in the case presented. Her anti-infective treatment was ineffective. Subsequently, TST and IGRA were positive, but the sputum acid-fast bacilli smear test was negative. No other tests related to sputum, so she started anti-tuberculosis treatment after a clinical diagnosis of pulmonary tuberculosis. The child’s clinical symptoms reduced after 2 months of therapy. Subsequently, the chest CT showed the lesion had progressed to miliary pulmonary tuberculosis. There was no history of exposure to drug-resistant TB patients. The patient’s compliance was good throughout the treatment. The symptoms resolved after the first phase of treatment, followed by a recurrence of the disease. The girl might initially be infected with wild-type resistant bacteria. The broad-spectrum antibacterial effect exerted by rifampicin could relieve clinical symptoms after the application of first-line anti-tuberculosis drugs. In addition, some strains might be effective with ethambutol and pyrazinamide. Another explanation is drug-susceptible and drug-resistant Mycobacterium tuberculosis in the patient. Initially, the sensitive tubercle bacilli was predominant. After conventional anti-tuberculosis treatment, drug-resistant tubercle bacilli gradually became dominant, causing an exacerbation of the disease. Yet, no DST evidence was available for this in the early stages of treatment, so it could not be confirmed.

Mycobacterium tuberculosis reaches the lungs in the bloodstream and becomes miliary pulmonary tuberculosis. Besides the lungs, tuberculosis bacteria can also spread to the lymph nodes, meninges, liver, spleen, and other organs throughout the body. When tuberculosis bacteria invade the nervous system, causing non-purulent bacterial inflammation of the meninges and involving the pia mater, arachnoid membranes, and brain parenchyma, it is called TBM, which is the most deadly type of tuberculosis. TBM is often secondary to tuberculosis foci in other parts of the body, especially hematogenous spread tuberculosis, so patients with miliary pulmonary tuberculosis should be screened in time to determine whether there is TBM and tuberculosis in other parts. In addition, we need to search for pathogenic evidence and get DST results to improve the accuracy of diagnosis. Unfortunately, this child did not carry out these tasks and only adjusted some medication and continued treatment. As a result, it was conceivable that the child developed fever, dizziness, headache, blurred vision, and other neurological symptoms again, at which time TBM was confirmed. The girl’s brain-enhanced MRI showed significant enhancement of the brain parenchyma, brain pools, and meninges, which is consistent with the imaging features of TBM [3, 9]. An Xpert MTB/RIF in CSF found Mycobacterium tuberculosis, with unknown drug resistance. Only 3 months later, we got phenotypic susceptibility results from the strains that were culture-positive for CSF, and the child was eventually diagnosed with pre-XDR TBM.

Besides the difficulty of diagnosis, drug-resistant tuberculosis has also been facing enormous challenges in treatment. WHO regularly updates corresponding application guidelines, demonstrating the rapid development of the field of drug-resistant tuberculosis and the importance that society attaches to drug-resistant tuberculosis. Guidelines published by the WHO endorse all-oral regimens to treat drug-resistant TB [10]. However, anti-TB regimens should be based on susceptibility results and patient-specific susceptibility results with fluoroquinolones, which play an important role in the treatment of drug-resistant TB. After over 40 years of exploration, two new agents (bedaquiline and delamanid) are available to treat MDR/XDR-TB [11, 12] and find new uses for older drugs such as linezolid. WHO divided anti-tuberculosis drugs into groups A, B, and C. Groups A and B, being all-oral drugs, from the core drugs of the all-oral chemotherapy regimen and are an essential basis for treatment [13]. An effective regimen should include at least four potentially effective anti-TB drugs, while the consolidation phase should include at least three potentially effective drugs. The WHO also recommends that treatment regimens for drug-resistant TBM be based on tuberculosis and for childhood tuberculosis in adults [14]. Several anti-TB drugs have different pharmacokinetics in children compared with adults, and some have poor CSF permeability because of the blood–brain barrier. Therefore, when developing a regimen for drug-resistant TBM, at least four effective drugs, including two or three with moderate CSF permeability, are necessary [15, 16]. The chemotherapy drugs selected by this child were all oral, but the DST showed resistance to fluoroquinolones, bedaquiline had poor CSF penetration with the limited data [17]. And delamanib was not yet available in China. Therefore, we selected drugs with good CSF permeability, such as linezolid, cycloserine, and pyrazinamide. Although adverse drug reactions occurred during treatment, we handled them promptly and without serious consequences, and the eventual outcome was satisfactory.

From the initial pneumonia to the clinical diagnosis of common tuberculosis and finally the diagnosis of drug-resistant TBM, the entire process was tortuous but finally got a good outcome. Throughout the process, we also found some limitations and learned some lessons. Limitations: (1) No repeated screening for bacteriology and DST results when considering tuberculosis. (2) No timely screening for spreading to other areas, especially the cranial area, in the presence of miliary pulmonary tuberculosis. These may cause a delay in the diagnosis and treatment of the disease, resulting in adverse outcomes such as neurological sequelae and life-threatening events.

We have also learned: (1) In the process of diagnosis and treatment of tuberculosis, we should constantly look for the bacteriology, get DST results as much as possible, and achieve an accurate diagnosis. It is in line with the WHO recommendations for TB diagnosis and treatment. (2) Patients with miliary pulmonary tuberculosis should be screened to determine whether there is tuberculosis in other parts, especially TBM, which is extremely fatal. 3) When planning a regime for drug-resistant TBM patients, it is necessary to give preference to drugs that can penetrate the blood–brain barrier and have high cerebrospinal fluid permeability and combine the specific conditions of patients with the DST results. 4) During the anti-tuberculosis treatment, we should closely monitor adverse drug reactions to avoid negative effects on the patient’s body and psychology because of severe adverse reactions.

In conclusion, the most harmful and severe type of TB is drug-resistant TBM, which is the most difficult to identify and cure. More people with drug-resistant TB will benefit from it when new technologies and medications. However, studies related to drug-resistant TBM in children are still limited, and staff in this specialty need to do more studies to provide the best diagnosis and treatment options for children with drug-resistant TBM.

## Data Availability

The datasets used and/or analyzed during the current study are available from the corresponding author on reasonable request.

## Abbreviations

TBM: Tuberculous meningitis
CSF: Cerebrospinal fluid
DST: Drug sensitivity test
CT: Computed tomography
MRI: Magnetic resonance imaging
TB: Tuberculosis
WHO: World Health Organization
IGRA: Interferon-Gamma Release Assay
TST: Tuberculin Skin Test
pre-XDR TBM: Pre-extensive drug-resistant
XDR-TB: Extensive drug-resistant TB
MDR-TB: Multidrug drug-resistant TB
